# Clinical Results After Single-fraction Radiosurgery for 1,002 Vestibular Schwannomas

**DOI:** 10.7759/cureus.6390

**Published:** 2019-12-16

**Authors:** Paul Y Windisch, Joerg-Christian Tonn, Christoph Fürweger, Berndt Wowra, Markus Kufeld, Christian Schichor, Alexander Muacevic

**Affiliations:** 1 Radiation Oncology, European CyberKnife Center, Munich, DEU; 2 Neurosurgery, Ludwig Maximilian University of Munich, Munich, DEU; 3 Medical Physics, European CyberKnife Center, Munich, DEU; 4 Oncology, European CyberKnife Center, Munich, DEU; 5 Neurosurgery, University Hospital of Munich, Munich, DEU; 6 Neurosurgery, Radiosurgery, European CyberKnife Center, Munich, DEU

**Keywords:** vestibular schwannoma, radiosurgery, acoustic neuroma, radiotherapy, neurofibromatosis

## Abstract

Background

Herein, we report clinical results for patients treated with stereotactic radiosurgery (SRS) for vestibular schwannomas (VS) over a period of 10 years.

Methods

Clinical data and imaging follow-up were stored in a database of 1,378 patients, with 1,384 VS treated consecutively between 2005 and 2018 and analyzed retrospectively. A total of 996 patients with 1,002 tumors with at least one year of follow-up were included for analysis.

Results

Median follow-up was 3·6 years (1-12·5 years). The three, five, and 10-year Kaplan-Meier estimated local tumor control was 96·6%, 92·3%, and 90·8%, respectively. The median hearing loss of the affected ear as compared to its healthy counterpart was 17 dB at treatment start and increased to 23 and 29 dB at one and five years. Six patients (0·6%) developed symptomatic hydrocephalus and underwent the placement of a ventriculoperitoneal shunt. In 30 patients (3·0%), trigeminal sensory dysfunction developed, five patients (0·5%) had a mild transient weakness, and nine patients (0·9%) had a permanent facial weakness (House-Brackmann Grade > II) after SRS.

Conclusion

Single fraction SRS proves to be highly effective and shows low treatment-related toxicity for VS. SRS should be considered a primary treatment option for small and middle-sized VS.

## Introduction

Stereotactic radiosurgery (SRS) becomes increasingly popular for mostly small vestibular schwannomas (VS) due to its treatment efficiency and ease of use as compared to surgical tumor resection. However, long-term data with reasonably large patient cohorts are missing and few quality-of-life evaluations after SRS for VS have been presented to date [1-2]. Moreover, most published series comprise smaller heterogeneous patient groups treated with inconsistent radiosurgical techniques and doses [3-5]. While there has been a tendency to treat increasingly smaller and sometimes asymptomatic tumors, concerns have been raised regarding long-term hearing toxicity and cases of suspected malignant transformation [6-7]. The purpose of this study was to analyze the functional outcome and the local tumor control after SRS for VS of a large patient group treated with the same technique in a dedicated treatment center over a period of 10 years.

## Materials and methods

The treatment records of 1,378 patients with 1,384 VS treated with CyberKnife-based SRS (Accuray Inc., Sunnyvale, CA) at the European CyberKnife Center in Munich between 2005 and 2018 were collected in a database for SRS [8]. CyberKnife is a frameless, image-guided robotic SRS system [9]. The therapeutic radiation is generated by a 6-MV compact linear accelerator mounted on a six-axis robotic manipulator. In a typical VS treatment, 100-200 non-isocentric, non-coplanar beams are directed at the tumor. Intra-fraction patient motion is compensated by the automatic adaptation of beam directions based on stereoscopic X-ray images of the patient’s skull acquired periodically during treatment. Patients who received SRS as a treatment for recurrence after previous radiotherapy were excluded. Two cases where the tumor was considered a surgery-induced metastasis and four cases where patients were treated in more than a single fraction were excluded as well. A total of 996 patients with 1,002 tumors had at least one year of follow-up after SRS and were included for analysis. Follow-ups consisted of a clinical examination and magnetic resonance imaging (MRI). Audiograms were recorded by otorhinolaryngologists elsewhere and added to our database during follow-up.

Follow-ups were performed after six months, every year for two years, and every two years thereafter. Tumor response was assessed by MRI. Shrinkage and no change in size were scored as a locally controlled disease. Increased size in two consecutive follow-ups was interpreted as a local recurrence.

Facial nerve palsy was assessed using the House-Brackmann (HB) score. Hearing function and ototoxicity were assessed using bilateral serial pure tone audiometry as described previously [10]. Only patients with testable hearing prior to SRS (defined as Gardner-Robertson Class 1-4) were included in the analysis.

First, to determine the overall hearing loss, bilateral serial pure tone audiometry was performed including the frequencies 0·5, 1, 2, 4, and 8 kHz. Then, the net hearing loss was calculated at each frequency as the difference between the hearing thresholds of the healthy ear and the affected ear. The mean of the net hearing loss values at the frequencies of 0·5, 1, 2, 4, and 8 kHz defined the overall hearing loss in decibels (dB). Hearing loss attributable to radiosurgery was calculated as the difference between the overall hearing loss at the time of radiosurgery and during follow-up. Worsening of hearing loss attributable to radiosurgery between SRS and follow-up by more than 20 dB was defined as ototoxicity.

Statistical analysis was performed with the Statistical Package for Social Sciences (SPSS) v. 23.0 (IBM SPSS Statistics, Armonk, NY) and Prism v. 8.0 (GraphPad, San Diego, CA). The significance of time to event data was assessed using the Cox proportional hazards model and the log-rank test. Variables tested for predictive significance concerning local recurrence were age, sex, side of the tumor, NF2 status, prior surgery, tumor volume, and radiosurgical prescription dose. In the case of a toxicity analysis, tumor recurrence was also included as a variable in the models and multivariate analysis was performed accordingly. Local control was plotted as a Kaplan-Meier survival curve for each variable of interest. Continuous variables were split into two groups at their respective median. All data was gathered in accordance with the World Medical Association Declaration of Helsinki.

## Results

Patient characteristics

Patient characteristics are depicted in Table 1. The median age at SRS was 55.1 years (range: 15.1 - 85.2 years) and the median follow-up was 3.6 years (1 - 12.5 years). All tumors were treated in a single fraction, with a median prescription dose of 13 Gy (11.5 - 15 Gy). The median prescription isodose line was 65% (55% - 80%). Tumors that had undergone surgical resection prior to SRS received a median dose of 13.5 Gy. While 827 tumors (82.5%) had not been treated previously, 175 tumors (17.5%) had undergone surgical resection. Of those 175 tumors, 39 received SRS due to subtotal resection while the remaining 136 schwannomas had recurred. Median tumor volume was 0.61 ccm (0.03 - 13.5 ccm). Thirty-one tumors (3.1%) were NF2-associated.

**Table 1:** Patient characteristics Numbers in parentheses denote ranges if not specified otherwise

Number of patients		996	
Number of tumors		1002	
Localization			
left		530	52.9%
right		472	47.1%
Sex			
male		459	46.1%
female		537	53.7%
Median age [yr]		55.1	(15.1 - 85.2)
Pretreatment			
none		827	82.2%
Surgery (Residual Tumor)		39	3.9%
Surgery (Local Recurrence)		136	13.5%
Follow-up			
Median [yr]		3.6	(1.0 - 12.5)
>= 1 yr		1002	
>= 3 yr		609	
>= 5 yr		321	
>= 10 yr		48	
NF2-associated tumors		31	3.1%
Median tumor volume [cc]		0.61	(0.03 - 13.5)
Median dose [Gy]		13	(11.5 - 15)
Median Isodose [%]		65	(55 - 80)

Tumor control

**Figure 1:**
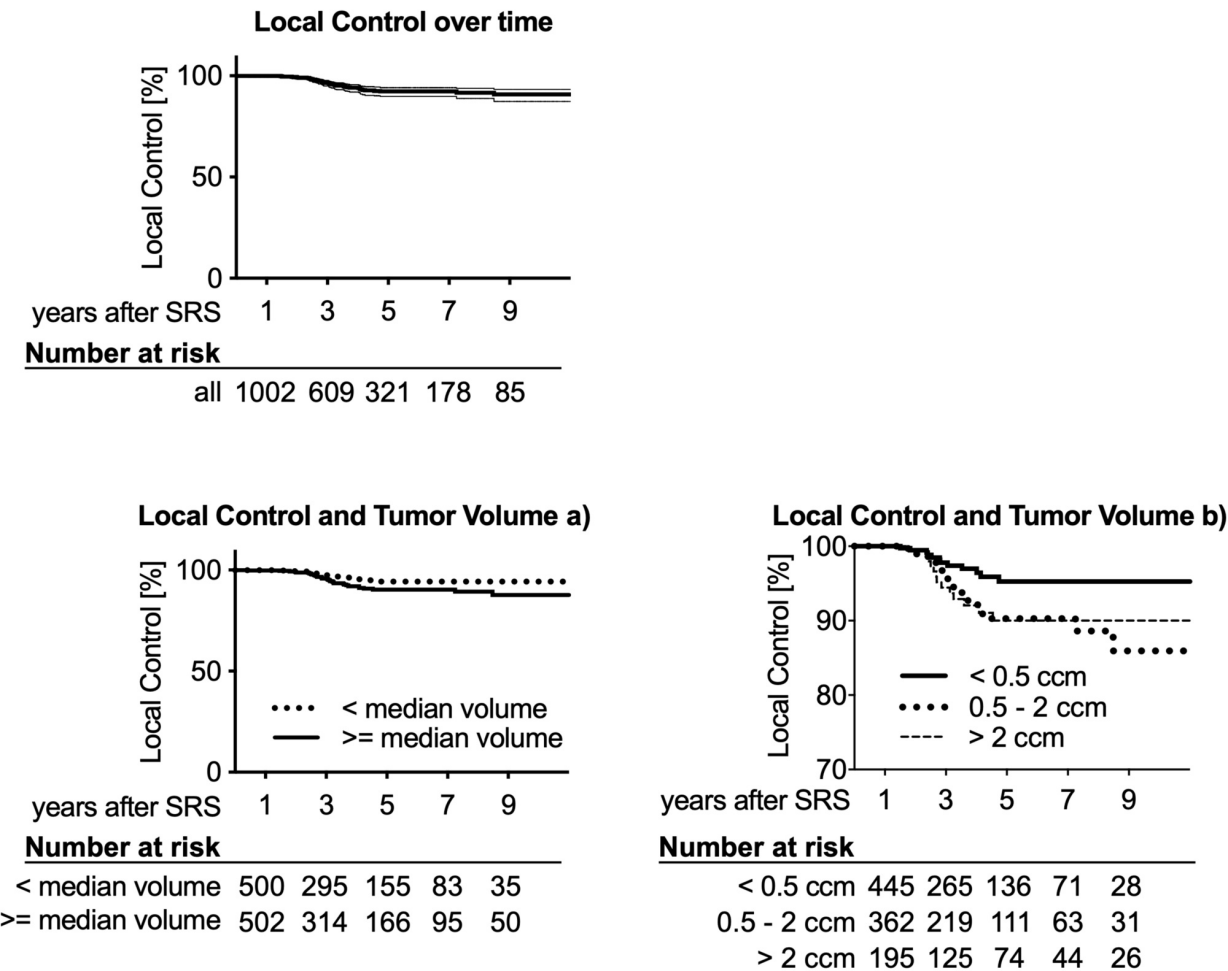
Kaplan-Meier estimates of local control over time The three, five, and 10-year local tumor control was 96.6%, 92.3%, and 90.8%, respectively. Thin lines in the ‘Local control over time’ plot indicate 95% CI intervals. The number of tumors at risk for each subgroup is depicted below each graph. Larger tumors are associated with significantly reduced local control.

Three, five, and 10-year follow-up data were available for 609, 321, and 48 tumors, respectively, showing Kaplan-Meier estimates for local control of 96.6% (95% CI: 94.9% - 97.7%), 92.3% (95% CI: 89.8% - 94.3%), and 90.8% (95% CI: 87.2% - 93.9%) (Figure 1).

**Figure 2:**
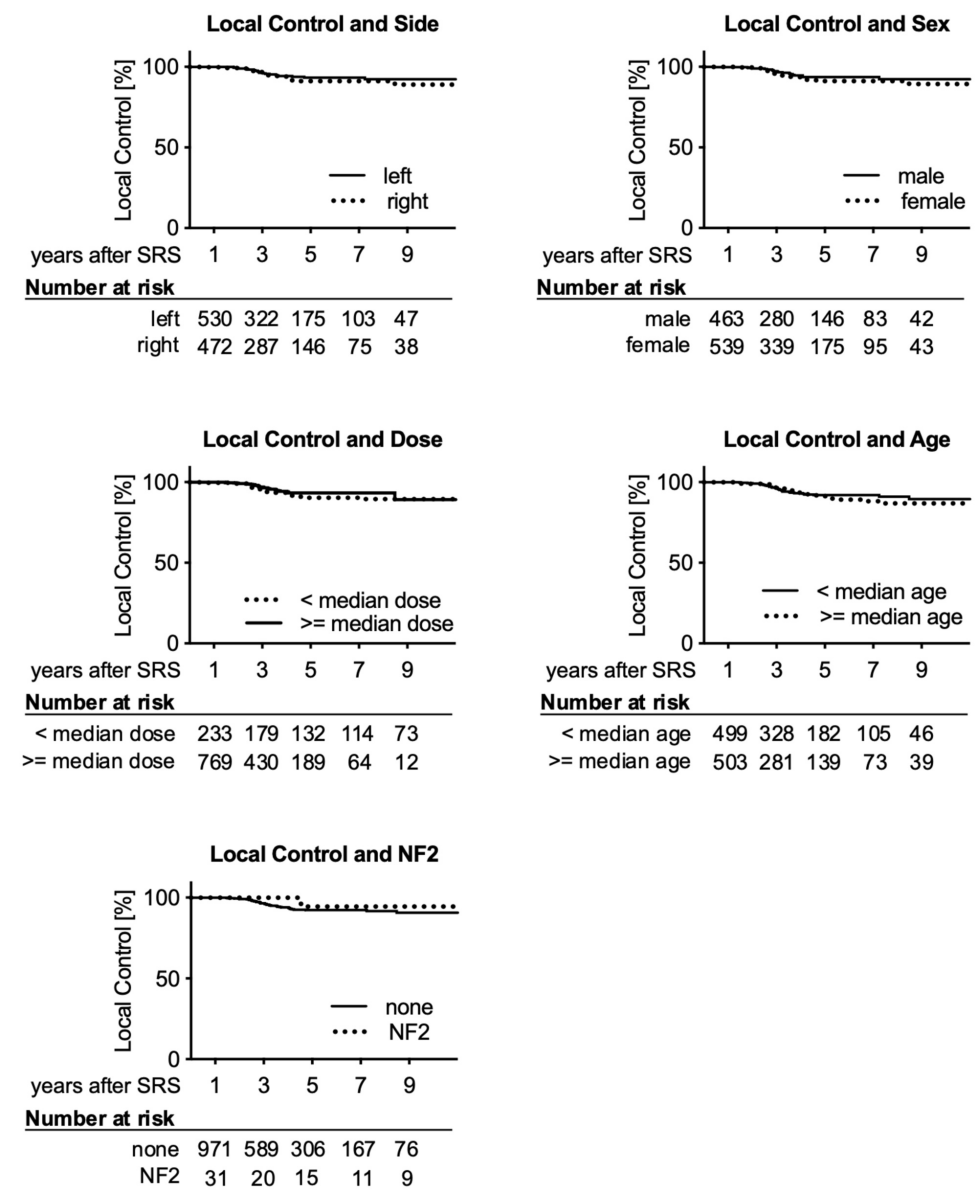
Kaplan-Meier estimates of local control over time Number of tumors at risk for each subgroup is depicted below each graph

Tumor volume was a significant predictor of local control, with larger volumes being associated with worse control in both the Cox proportional hazards model and the log-rank test (Table 2). When splitting the tumors into two groups at the median (0.61 ccm), Kaplan-Meier estimated local control at three, five, and 10 years was 97.4%, 94.4%, and 94.4% for smaller and 95.7%, 90.3%, and 87.7% for larger tumors (Figure 1). Age, sex, side, NF2 status, and dose were not predictive of local control (Figure 2). Surgery prior to SRS was only significant in the univariate analysis.

**Table 2:** Cox proportional hazards model predicting local control reveals tumor volume as the only significant predictive variable Numbers in parentheses denote ranges if not specified otherwise

	Hazard ratio	95% CI	p	Log-rank
Age (years)	0.99	0.97 - 1.01	0.169	0.414
Sex (f/m)	1.36	0.76 - 2.42	0.299	0.217
Side (r/l)	1.23	0.70 - 2.17	0.464	0.309
NF2	0.25	0.03 - 1.97	0.19	0.486
Surgery	1.72	0.93 - 3.16	0.086	0.035
Tumor vol (cc)	1.16	1.02 - 1.33	0.033	0.026
Dmin (Gy)	0.82	0.65 - 1.02	0.072	0.262

Creating three subgroups of tumor volumes (< 0.5 ccm, 0.5 - 2 ccm, > 2 ccm) revealed that the smallest tumors showed significantly improved local control as compared to both, the middle-sized (p = 0.0153) and larger schwannomas (p = 0.038), which, in turn, did not differ significantly from each other.

Of the 49 patients who experienced tumor recurrence, 13 received an additional CyberKnife treatment at a median of 3.2 years (2 - 8.5 years) after initial SRS while 16 underwent surgery. The remaining 20 cases were either very recent so that the additional therapy had not been documented at the time of this study or the patients were lost to follow-up. The median follow-up for SRS retreatment was 4.6 years (range: 0.5 - 8.1 years). Local control was achieved in all cases while no grave toxicity was observed. The median volume of the re-irradiated tumors was 1.94 cc (range: 0.59 - 5.34 cc).

Toxicity

The HB score prior to treatment was available for 997 tumors. Of the 943 cases with good facial function (HB grades I-II) before SRS, 14 (1.5%) experienced worsening to HB grade III-V, which was transient in five cases. In six of the nine cases where worsening of facial nerve function was permanent, the tumor had recurred and three of these recurrences had already been treated with surgical resection. No patient experienced total facial nerve palsy (HB VI) following SRS.

Of the 54 patients with HB grade III-VI prior to SRS, four patients (7.4%) experienced an improvement of facial nerve function to HB I-II, which was permanent in all cases.

Valid audiograms prior to treatment and at one year (6 - 18 months) post-treatment were available for 210 patients. Fifty-five patients had valid pre-treatment and five-year (48 - 72 months) post-treatment audiograms. Median hearing loss prior to SRS was 17 dB and increased to 23 dB at one year and 29 dB at five years post-treatment.

At one year, 63 ears (30%) experienced an improvement in hearing as compared to the healthy ear while five patients (2.4%) had no change in hearing deficit and 142 patients (67.6%) experienced worsening. However, only 23 of these patients (10.9%) experienced ototoxicity as defined by an increase of hearing loss >= 20 dB. Results of audiograms at one-year post-SRS are depicted in Table 3.

**Table 3:** Hearing toxicity at year one post-SRS Numbers in parentheses denote ranges if not specified otherwise SRS: stereotactic radiosurgery

Number of patients			210	
Median HL pre-treatment [dB]			17	(-45 - 72)
Median HL at 1 year [dB]			23	(-40 - 90)
Patients with ototoxicity (HL >= 20 dB)			23	10.9%
Patients with improved hearing			63	30.0%
Patients with worsened hearing			142	67.7%
Patients with unchanged hearing			5	2.4%

At five years, 12 patients (22.8%) experienced an improvement in hearing as compared to the healthy ear while the remaining 43 patients (78.2%) experienced worsening. However, only 13 (23.6%) of these patients experienced ototoxicity as defined by an increase of hearing loss >= 20 dB post-SRS. The results of audiograms at five years post-SRS are depicted in Table 4.

**Table 4:** Hearing toxicity at year five post-SRS Numbers in parentheses denote ranges if not specified otherwise.\ HL: hearing loss

Number of patients			55
Median HL pre-treatment [dB]			17	(-64 - 53)
Median HL at 5 years [dB]			29	(-7 - 91)
Patients with ototoxicity (HL >= 20 dB)			13	23.6%
Patients with improved hearing			12	21.5%
Patients with worsened hearing			43	78.2%
Patients with unchanged hearing			0	0.0%

Two patients had seizures during their follow-up period, without any hints suggesting an association with the tumor or the treatment.

Treatment-associated hydrocephalus requiring shunt implantation could be observed in five patients (0.5%). One patient developed hydrocephalus due to local recurrence and received shunt implantation combined with microsurgical resection. The median tumor volume of patients with treatment-associated hydrocephalus requiring shunt implantation was 3.38 ccm (0.27 - 7.88 ccm) with five of the six tumors being larger than 2.4 ccm.

Thirty-one patients (3.1%) who reported no trigeminal sensory dysfunction at treatment start presented symptoms during follow-up examinations. However, these were permanent in only five patients (0.5%) as defined by having trigeminal sensory dysfunction at each patient’s most recent respective follow-up.

No case of malignant tumor transformation was observed.

## Discussion

Local tumor control

As most of the studies on the long-term safety and efficacy of SRS for VS are based on different treatment technologies, such as the Gamma Knife (Elekta, Stockholm, Sweden), this study, to the best of our knowledge, analyzes the largest patient collectives treated exclusively with CyberKnife, as well as the largest collective treated with SRS in general. The findings concerning local control > 90% even 10 years after treatment are in line with other studies on the long-term efficacy of SRS for VS using Gamma Knife radiosurgery [11]. Given that, in some cases, local recurrence was diagnosed within less than two years following treatment, the actual tumor control might be even higher, as pseudoprogression is a frequent cause of volume change after SRS for VS, especially in the first 24 months post-treatment [12].

The association of reduced local control with increased tumor volumes has been the subject of ongoing discussion [13]. Analyzing local control for tumors smaller and larger than the median volume (0.61 ccm) resulted in local control of 97.4%, 94.4%, and 94.4% at three, five and 10 years for the smaller and 95.7%, 90.3%, and 87.7% for the larger group, which corresponds to a recent study by Ruess et al. where, for a group of 335 patients with a median tumor volume of 1.1 ccm, local control was 89% and 87% at five and 10 years, respectively [14]. These differences, especially in long-term control, should be considered when deciding to place smaller tumors under surveillance.

The finding that prior surgery was a significant factor predicting local control in the univariate, but not in the multivariate, analysis might be due to the fact that tumors, which had undergone surgery prior to SRS, had significantly larger volumes (median volume 1.31 ccm, p < 0.0001).

The missing dose-effect on tumor control that has been reported by previous publications, including one from this institution, and could be explained by the narrow dose range as 963 of 1,002 tumors were irradiated with 12.5 - 13.5 Gy [10,15].

Contrary to most research on SRS for VS, NF2 was not associated with reduced local control. Given the limited number of NF2-associated tumors (n = 31) in this study (but also in many other publications), drawing definite conclusions from this finding is difficult [16-18]. However, the median tumor volume for NF2-associated tumors was 0.82 ccm, which is considerably smaller than in many existing publications [18]. Mathieu et al. report local control rates of 85% and 81% at five and 10 years following Gamma Knife radiosurgery of NF2-associated VS for 74 tumors with a mean volume of 5.7 ccm [17]. One could, therefore, hypothesize that the effect of reduced local control associated with NF2 loses predictive significance once the tumors are irradiated while at a sufficiently small volume. As many studies on NF2-associated schwannomas already report tumor volume as a predictor of local control, subgroup analyses of these collectives could answer the question, whether treatment of small NF2-associated tumors may result in equally good local control rates as treatment of sporadic VS [16-17].

Retreatment

In several cases where the initial SRS treatment could not stop tumor growth, patients were suitable to receive SRS retreatment. While there is still very little data on retreatment with SRS for VS (which makes it difficult to assess the risk associated with the accumulation of radiation dose), retreatment seems to be a safe and effective option. Others have reported good tumor control upon Gamma Knife retreatment as well, but noted a slightly higher rate of facial nerve toxicity, at least compared to the patient group that received SRS as the initial treatment in this study [19].

Hearing

A meta-analysis of hearing outcomes following SRS for VS and a study by Santa Maria et al. comprising 344 patients with audiograms and more than three years of follow-up reported hearing preservation of 51% and 50%, respectively, at three years post-treatment [7,20].

Even though ototoxicity, as defined in this study, occurred in only 23.2% of cases, there is a risk of underestimating the extent of ototoxicity, as patients who have no remaining hearing on the treated (or both) ears might stop doing audiograms as part of their follow-up and as a reduction in hearing capacity of the healthy ear reduces the difference between healthy and affected ear. This should be considered when irradiating very small tumors that have not shown significant growth in an attempt to save the patient’s hearing.

However, if growth is present, the hearing function has been reported to decline fairly quickly, with patients often losing serviceable hearing within the first five years in cases where the tumor has been left untreated [21].

Therefore, in the case of small tumors and good hearing, we suggest monitoring the hearing function closely with six-month intervals and suggest treatment only when a hearing decline can be documented and/or the tumor is growing.

Facial nerve toxicity

While facial nerve toxicity is rare as compared to hearing toxicity, it can severely impact the patient’s quality of life. A meta-analysis covering 1,908 patients by Yang et al. described an association between lower marginal doses of 13 Gy or less and reduced facial nerve toxicity for Gamma Knife radiosurgery [22]. However, reducing the dose may result in tumor recurrence, which was seen in the majority of cases with facial nerve toxicity in this study. Additionally, increased tumor volume was associated with higher rates of facial nerve toxicity. Overall, the authors report a facial nerve preservation rate of 96.2%.

In a study of facial nerve function after translabyrinthine vestibular schwannoma surgery on 392 patients, 81% had HB grade I-II one year after surgery while 12 patients experienced total facial nerve palsy (HB grade VI) [23].

Falcioni et al. reported anatomical interruption of the facial nerve in 48 out of 1151 cases. Thirty-five percent of the remaining cases where the facial nerve could be preserved had postoperative HB grade III or worse. Smaller tumors had a better facial nerve outcome with postoperative HB grade III or worse occurring in 14% of 444 patients with tumor diameters of less than 1 cm [24].

Hydrocephalus

A study by Lee et al. reported a hydrocephalus incidence of 4.1% for 702 patients treated with Gamma Knife radiosurgery and found age, tumor origin, and tumor volume as significant predictors [25]. The higher incidence could be due to a higher mean tumor volume of 3.6 ccm as compared to 1.25 ccm in this study. The median age of the patients who developed hydrocephalus in this study was 55.9 years (48.4 - 74.6 years), only marginally higher than the median age of the whole collective. Hydrocephalus is also a rare complication when treating VS surgically.

## Conclusions

SRS is a safe and effective treatment option for treating VS with tolerable toxicities. Though additional and particularly prospective studies are desirable, SRS should be considered a primary treatment option for small and middle-sized vestibular schwannomas.
